# Disseminated *Talaromyces marneffei* in newly diagnosed HIV patient

**DOI:** 10.1002/jha2.460

**Published:** 2022-04-28

**Authors:** Yingcai Huang, Haiyan Cui, Yanxia Chen, Jinlin Liu

**Affiliations:** ^1^ Department of Clinical Laboratory Longgang District People's Hospital of Shenzhen & The Second Affiliated Hospital of the Chinese University of Hong Kong Shenzhen China; ^2^ Center for General Practice Medicine Department of Rheumatology and Immunology Zhejiang Provincial People's Hospital (Affiliated People's Hospital, Hangzhou Medical College) Hangzhou China; ^3^ Laboratory Medicine Center Department of Clinical Laboratory Zhejiang Provincial People's Hospital (Affiliated People's Hospital, Hangzhou Medical College) Hangzhou China

1

A 31‐year‐old Chinese man referred to the haematology clinic, was presented with recurrent fever for 19 days and two enlarged lymph nodes on the left neck. Past medical history was unremarkable. Laboratory data revealed white blood cell (WBC) count 9.81 × 10^9^/L (neutrophilic band granulocyte 67%, neutrophilic myelocyte 11%, neutrophilic metamyelocyte 7%, polymorphonuclear neutrophil 6%, lymphocyte 5%, eosinophil 1%, monocyte 2%, basophil 1% and nucleated red blood cell 18/100 WBC), haemoglobin 77 g/L and platelet count 41 × 10^9^/L. The peripheral blood and bone marrow aspirate smear revealed small round‐to‐ovoid yeast cells located predominantly within or surrounding the neutrophil (top left, middle and right, 1000×, Wright‐Giemsa staining) Figure. The blood culture showed the fungus grown in a mould form arranged in a mulberry‐like pattern and produced a soluble red pigment that diffuses into the sabouraud medium (bottom left). Lactophenol cotton blue staining confirmed numerous yeast‐like organisms by the microscopic morphology (bottom middle, 400×). Fungus staining also showed numerous blue yeast‐like organisms by fluorescence microscope (bottom right, 400×).


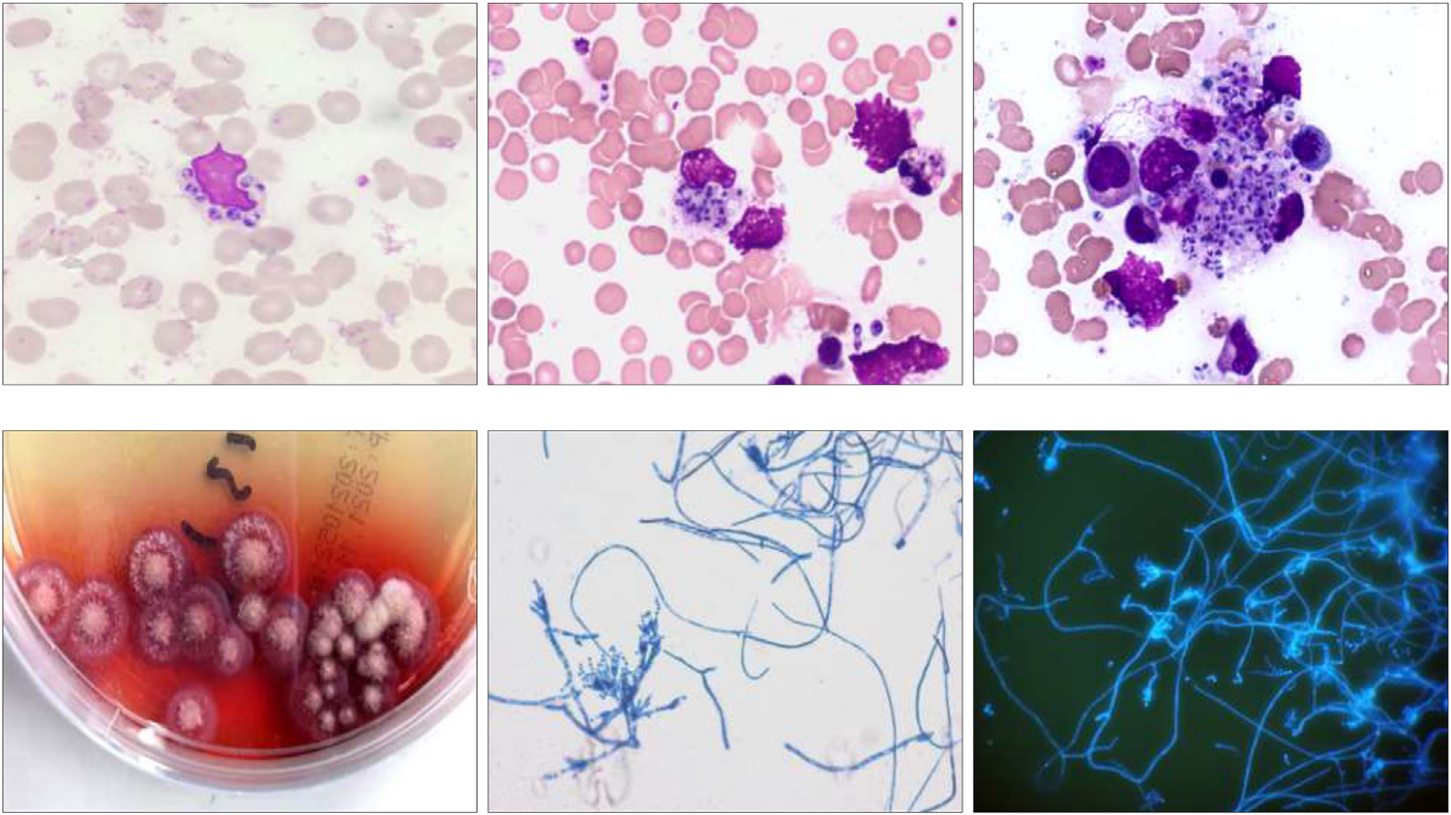


The patient was subsequently diagnosed with AIDS having HIV antibody, and the blood cultures showed the growth of *Talaromyces marneffei*, a dimorphic fungus that is an important opportunistic pathogen. After treatment with anti‐fungus and anti‐HIV drug, this patient had significantly improved and discharged.

## CONFLICT OF INTEREST

The authors declared that they have no conflict of interest.

## AUTHOR CONTRIBUTIONS

Yingcai Huang provided the haematology picture and wrote the manuscript. Haiyan Cui provided the microbiology picture. Yanxia Chen analysed the data. Jinlin Liu wrote the manuscript. All authors reviewed this manuscript and agreed to submit it.

## ETHICS STATEMENT

This study was approved by the ethical committee of Longgang District People's Hospital of Shenzhen & The Second Affiliated Hospital of the Chinese University of Hong Kong (No. 2022007).

## PATIENT CONSENT STATEMENT

This study did not involve any personal information, but only reports the laboratory data. Therefore, the patient consent was waived, and this waive was approved by the ethical committee of Longgang District People's Hospital of Shenzhen & The Second Affiliated Hospital of the Chinese University of Hong Kong.

